# Hantaviruses at the human-animal-environment interface: a One Health review of rodent reservoirs, surveillance, and preparedness

**DOI:** 10.3389/fvets.2026.1962397

**Published:** 2026-10-30

**Authors:** Muhammad Inam, Hamid Khan

**Affiliations:** 1 School of Medicine, Qilu Institute of Technology, Jinan, China; 2 Institute of Brain Science and Brain-Inspired Research, Shandong First Medical University and Shandong Academy of Medical Sciences, Jinan, China

**Keywords:** Andes virus, hantavirus, occupational exposure, One Health, orthohantavirus, preparedness, rodent reservoir, surveillance

## Abstract

Hantaviruses maintained in small mammals cause hemorrhagic fever with renal syndrome and hantavirus cardiopulmonary syndrome. Exposure usually arises from contact with infected rodent excreta, so prevention depends on understanding reservoir ecology, human activities, and the settings in which they intersect. This structured narrative review synthesizes evidence on host relationships, exposure, clinical and laboratory recognition, genomic investigation, and One Health surveillance across several regions. We propose three risk-proportionate tiers: baseline readiness where evidence is sparse, enhanced investigation after a credible signal, and sustained integrated surveillance where transmission is established. Five shared functions are scaled across tiers: reservoir sampling, clinical recognition and referral testing, molecular characterization, occupational and environmental assessment, and cross-sector data review. Published outbreaks in Croatia and long-running surveillance in Panama illustrate how the decision logic could be applied, while the 2026 Andes virus cruise-ship event highlights the need for diagnostics and contact management. The framework provides a practical way to plan surveillance with limited resources, but its triggers, workload, and impact remain to be evaluated prospectively.

## 1 Introduction

Hantaviruses are enveloped, three-segmented, negative-sense RNA viruses in the family Hantaviridae, order Elliovirales. Rodent-associated orthohantaviruses cause hemorrhagic fever with renal syndrome (HFRS) across much of Eurasia and hantavirus cardiopulmonary syndrome (HCPS) in the Americas (1–3). The transmission cycle brings clinical care into contact with wildlife ecology, occupational health, veterinary practice, and laboratory biosafety.

The defining One Health feature of hantavirus epidemiology is that the maintenance cycle operates entirely outside health services. Reservoir abundance and species composition, food availability, rainfall, temperature, land use, agricultural practice, housing quality, waste management and human behavior together determine whether infected animals contaminate spaces that people use (1, 4, 5). Humans are usually exposed by inhaling aerosolized particles from rodent urine, feces, or saliva, or through direct contact with contaminated material. Prevention therefore depends on recognizing and modifying ecological and occupational interfaces before a patient reaches clinical care. A systematic review of land cover and land use found agricultural land use positively associated with human infection risk in China and Brazil and forest cover positively associated with incidence in Europe, while associations with built-up areas differed in direction between studies (6).

Surveillance coverage differs markedly between regions. Some countries have paired human case reporting with reservoir monitoring for decades, while others rely on a few serosurveys or small wildlife studies, and some have no structured reservoir data at all (7–9). This makes country comparisons difficult: few reported cases may indicate genuinely low transmission, limited diagnostic access, missed cases, or simply insufficient surveillance. A meta-analysis of small mammals across Southeast Asia estimated a pooled hantavirus prevalence of 6.07% among 11,806 animals, with the highest estimates in Indonesia and Singapore, in a region where human diagnostic recognition remains limited (8). Work in Cambodia detected orthohantavirus RNA in 3.3% of 750 rodents and IgG seropositivity in 10.0% of people sampled at the same sites, with the highest rodent infection rates at urban rather than rural interfaces (9).

Attention to these questions has been renewed by the 2026 multi-country Andes virus (ANDV) event linked to an expedition cruise ship, which produced 13 cases and three deaths, contact tracing across more than 30 countries and territories, and a sequence of international notifications before it was declared over in July 2026 (10). The event does not indicate that hantaviruses have become more widespread. It illustrates how a rare zoonosis moving through a mobile and medically constrained exposure network can rapidly test diagnostic referral pathways and risk communication in settings without endemic transmission.

Recent field surveys, systematic reviews, and outbreak reports from 2024 to 2026 have expanded the evidence base on host range, land use, geographic surveillance gaps, and international response (6–10). Yet descriptive One Health reviews seldom specify how a low-capacity setting should move from baseline sampling to an investigation, or when an enhanced response can safely be reduced (11, 12). This review brings together reservoir biology, exposure, diagnostics, and surveillance to propose explicit, locally adaptable decision steps. The framework is an operational hypothesis, not an established standard.

## 2 Scope and evidence framing

### 2.1 Review design and information sources

This structured narrative review brings together evidence on hantavirus reservoirs, human exposure, diagnosis, and One Health surveillance. We searched literature using PubMed, Scopus, and Web of Science, supplemented by Google Scholar, reference lists, and reports from public health agencies. The searches were updated in August 2026. Studies published from 2000 onward received particular attention, while earlier work was retained when it remained foundational to the biology or clinical picture.

Searches combined “hantavirus” or “orthohantavirus” with terms for five related domains: reservoir hosts and ecology; occupational, veterinary and household exposure; serology, molecular diagnosis and genomic epidemiology; surveillance, land use and climate; and Andes virus outbreaks and contact management. Terms and combinations were adapted to each database and refined as the review developed. The purpose was to examine evidence relevant to practical surveillance decisions across different epidemiological settings.

### 2.2 Evidence eligibility and selection

Studies were retained when they directly informed one or more areas addressed in this review, including reservoir ecology and host relationships, animal-to-human exposure, human epidemiology, veterinary or occupational risk, diagnostic and genomic methods, environmental and land-use influences, surveillance, and preparedness. For specific epidemiological or laboratory statements, we relied mainly on peer-reviewed primary research; systematic and narrative reviews were used to place individual findings in a broader context. We also used guidance and outbreak reports from WHO, CDC, ECDC, and similar public or animal health agencies when these sources provided operational information not available in journal articles. We generally excluded papers devoted only to treatment or molecular mechanisms with no clear relevance to exposure or One Health surveillance, duplicate or substantially overlapping reports, studies with too little methodological detail to interpret, conference abstracts, and non-authoritative web sources.

### 2.3 Evidence synthesis and framework development

Priority was given to primary epidemiological, virological, reservoir, genomic, diagnostic and occupational studies, with systematic reviews and authoritative narrative reviews used for broader contextual statements. The synthesis was organized around five recurring questions: which animals maintain infection, where human exposure occurs, which populations are most exposed, how infection can be reliably detected, and how animal, human and environmental information can be combined into surveillance and prevention (11, 13, 14).

Because the literature considered differed substantially in study design, host species, geographic setting, diagnostic method, surveillance intensity, and reported outcomes, quantitative pooling was not considered appropriate. Findings were synthesized narratively across epidemiological settings, with particular attention to consistency, directness of evidence, methodological clarity, and operational relevance. Greater interpretive weight was given to longitudinal surveillance, well-characterized reservoir studies, linked human-animal investigations, molecularly confirmed outbreaks, and studies with clearly described sampling and diagnostic methods. The tiered surveillance framework was developed from recurring operational needs identified across these evidence domains and should be interpreted as an evidence-informed proposal rather than a formally validated guideline.

We considered five dimensions when interpreting a study: relevance to the question; reliable identification of the host and infection; a clear sampling denominator, time frame, and assay; a longitudinal or linked human-animal design when needed to support an inference; and agreement or disagreement with findings from other settings. A serological result without supporting exposure or molecular data was not taken as evidence of current transmission. These considerations informed the narrative synthesis rather than a formal quality score. Table 1 illustrates the kinds of evidence and methodological constraints that shaped the proposed surveillance framework.

**Table 1:** Selected evidence informing the design of integrated hantavirus surveillance.

Study and region	Design and period	Population	Sample size	Assay or source	Principal finding	Evidence domain	Surveillance implication	Key limitation
Goodfellow et al. (18) New Mexico	Field survey, 2019–2023; archived specimens	Small mammals	1,561 total; 255 at repeat sites	Tissue RT-qPCR; virus isolation	415/1,561 SNV RNA positive (26.6%); 91/255 at repeat sites	Reservoir hosts	Sample multiple species and revisit sites	Animal detection does not quantify human risk
Dubrulle et al. (25) Madagascar	Land-use survey	*Rattus rattus*	794 rats	Viral RNA testing	75/794 RNA positive (9.5%)	Reservoir hosts	Target sampling by host and habitat	Association does not establish human risk
Guillebaud et al. (9) Cambodia	Two-season survey, 2020 and 2022	Rodents; people	750 rodents; 788 people	Rodent RT-PCR; human IgG	25/750 RNA positive (3.3%); 79/788 IgG positive (10.0%)	Exposure settings	Pair animal and human sampling at shared sites	IgG does not confirm current infection or direct linkage
Guo et al. (8) Southeast Asia	Systematic review and meta-analysis	Small mammals	28 studies; 11,806 animals	Random-effects meta-analysis	Pooled prevalence 6.07%; I^2^ = 97.6%	Reservoir hosts	Tailor sampling to local hosts and assays	High heterogeneity precludes a universal threshold
Rončević Filipović et al. (33) Croatia	Outbreak report, 2021	Human cases	334 notified; 254 at one center	Clinical and notification records	14.6% of center patients were not notified	Reliable detection	Audit notification against clinical records	Single-center gap is not a national estimate
Armién et al. (34) Panama	National surveillance, 1999–2019	Human cases	712 cases	Case and laboratory surveillance	Endemic foci and annual variation identified	Data integration	Use long-term records to target diagnostics	Does not validate proposed tier triggers
Wang et al. (35) Taiwan	Feeder-rat farm investigation, 2022	Workers; feeder rats	3 workers; 8 rats	Worker testing; rat IgG	2 acute and 1 earlier worker infection; 3/8 rats IgG positive	At-risk populations	Coordinate worker and colony monitoring	No rat RT-PCR; only eight rats tested

The five evidence domains correspond to the guiding questions in Section 2.3. Surveillance implications synthesize each study’s contribution; percentages are not directly comparable because populations and assays differ.

### 2.4 Terminology and interpretive principles

Terminology is used deliberately throughout. Hantavirus refers to the group, while orthohantavirus is used where taxonomic precision is required. We distinguish reservoir hosts, in which infection is persistent and largely asymptomatic, from incidental hosts, and we distinguish serological evidence of past exposure from molecular detection of viral RNA, reservoir infection from human exposure, and human exposure from confirmed disease. Antibody detection in a rodent or a person is not by itself evidence of current transmission, and a positive rodent does not establish human disease risk in the absence of a plausible exposure pathway.

One interpretive principle applies throughout: absence of published surveillance is not equivalent to absence of infection. Evidence-sparse settings are therefore discussed in terms of surveillance gaps and preparedness needs rather than assumed local transmission or assumed local safety. The distinction matters particularly for neglected zoonoses, where diagnostic capacity and small-mammal sampling tend to be concentrated in a small number of research centers.

## 3 Hantavirus biology and disease spectrum

The genome comprises small (S), medium (M) and large (L) segments encoding the nucleocapsid protein, the Gn/Gc glycoprotein precursor and the RNA-dependent RNA polymerase, respectively (15). Genetic diversity arises through mutation, reassortment, prolonged host association and occasional host-switching events (16, 17).

Many hantaviruses show strong evolutionary association with particular rodent or small-mammal hosts, but the relationship is more dynamic than a strict one-virus-one-host model (16, 17). Field data increasingly support this. A survey of small-mammal communities in New Mexico detected Sin Nombre virus (SNV) in 27% of animals sampled and recovered infectious virus from tissues and feces of species other than the recognized primary reservoir, indicating that alternative and previously unrecognized reservoirs can both maintain and shed the virus (18). Surveillance designs that sample only the expected host species may therefore underestimate both prevalence and the range of species contributing to environmental contamination.

Old World orthohantaviruses, including Hantaan virus (HTNV), Seoul virus (SEOV), Dobrava-Belgrade virus (DOBV) and Puumala virus (PUUV), are associated primarily with HFRS. Severity ranges from the comparatively mild nephropathia epidemica associated with PUUV to hemorrhage, hypotension, and acute kidney injury (2, 3, 19). New World viruses, notably Sin Nombre virus (SNV) and Andes virus (ANDV), are associated predominantly with HCPS, which can progress rapidly to pulmonary edema and cardiovascular compromise (1, 2). Reported case fatality depends on the virus, the case definition, ascertainment period, and access to critical care. SEOV infection may be mild and can be missed without appropriate testing (20).

Table 2 places representative virus-host relationships beside their human syndromes and exposure settings. Its host associations are illustrative and should not be used to rule out infection in other small mammals.

**Table 2:** Major pathogenic hantaviruses, principal reservoir associations, main human syndromes and One Health relevance.

Virus (abbreviation)	Principal reservoir association	Main human syndrome	One Health relevance
Hantaan virus (HTNV)	*Apodemus agrarius* and related field mice	HFRS, often moderate to severe	Agricultural landscapes, seasonal field-rodent dynamics and occupational exposure; multiple genotypes co-circulate in endemic areas.
Seoul virus (SEOV)	*Rattus norvegicus* and related commensal rats	HFRS, usually milder and easily missed	Urban, port, farm, laboratory-rat, feeder-rodent and pet-rat settings; reservoir is globally distributed and infected rats are clinically normal.
Puumala virus (PUUV)	*Myodes glareolus* (bank vole)	Milder HFRS (nephropathia epidemica)	Vole population cycles, forest cover and mast years shape exposure; under-notification is documented even where capacity exists.
Dobrava-Belgrade virus (DOBV)	*Apodemus* spp.	HFRS, severity varies by genotype	Rural and forest habitat use; genotype identification requires molecular confirmation rather than serology alone.
Sin Nombre virus (SNV) and related New World viruses	*Peromyscus* spp. and other sigmodontine or neotomine rodents	HCPS	Reservoir population surges, peri-domestic infestation and disturbance of enclosed contaminated spaces; recent work shows circulation in multiple sympatric species.
Andes virus (ANDV)	*Oligoryzomys* spp.	HCPS	Reservoir ecology plus documented person-to-person transmission, which adds infection control and contact management to the usual reservoir-focused response.

HFRS, hemorrhagic fever with renal syndrome; HCPS, hantavirus cardiopulmonary syndrome.

Much of the tissue injury appears to reflect an abnormal host response, increased vascular permeability, and platelet and endothelial dysfunction rather than widespread direct viral cytolysis (19, 21, 22). Reservoir rodents, by contrast, generally remain outwardly healthy despite persistent infection, allowing them to maintain and shed virus over long periods (23). Person-to-person spread is uncommon for hantaviruses. ANDV is the important exception, with transmission documented after close, prolonged contact, so infection-control measures become relevant in a way they usually are not for other hantaviruses (2, 10).

## 4 Reservoir ecology and routes of animal-associated exposure

Human-pathogenic hantaviruses are maintained mainly in rodents, although related lineages also occur in shrews, moles, and bats (16, 17). Host associations help define geographic distribution, but surveillance is often more informative when risk is considered by setting rather than host species alone. Exposure arises where infected small mammals, contaminated material, and human activity overlap.

Most human infections are acquired indirectly from contaminated dust, bedding, grain, surfaces, or enclosed spaces. Risk is greatest during activities that disturb dried excreta, such as sweeping, clearing storage rooms, handling feed, disturbing nests, opening buildings that have been closed for long periods, or cleaning cages. Bites and direct handling contribute less often but are documented, particularly in occupational settings (1, 20, 24).

Agricultural and peri-domestic environments deserve particular attention. Grain stores, feed warehouses, poultry houses, livestock units, barns and farm outbuildings offer both food and shelter to commensal and field rodents. Urban interfaces include informal settlements, sewers, food markets, waste sites, transport hubs and older buildings with persistent infestation. Ecological work outside the classical endemic regions supports the emphasis on setting: in northeastern Madagascar, hantavirus RNA related to Anjozorobe virus was detected in 9.5% of sampled *Rattus rattus* across a land-use gradient, with the highest infection probability in flooded rice fields and the lowest in villages and relatively intact forest (25). Findings of this kind are locally specific and should not be generalized, but they illustrate why exposure assessment has to be anchored in how people actually use land.

Laboratory-animal colonies, feeder-rodent production and pet-rat networks form a distinct and well-documented pathway. SEOV circulates among clinically normal *Rattus norvegicus*, which shed virus chronically in urine, feces and saliva, so infection remains invisible without deliberate testing (20, 23, 26). Reported human infections have been linked to breeding colonies and pet-rat supply chains in several countries, and molecular comparisons have matched viruses from patients with those recovered from their own rats (20, 26).

Markets, feed-storage facilities, ports, and waste-management systems are other settings where veterinary and municipal responsibilities overlap, as routine control work already involves contact with rodents (27, 28). Community programs show that structured rodent monitoring can be added to existing control activities in disadvantaged urban areas rather than run as a separate research project (28). Control work should still minimize disturbance of heavily contaminated material and the resulting aerosol exposure.

Field work with wildlife creates a somewhat different pattern of exposure. Trapping, handling, necropsy, specimen processing, and work inside enclosed field structures can expose staff directly to infected animals and contaminated material (13, 24). Training in personal protective equipment, standardized trapping protocols, decontamination and exposure reporting should be built into wildlife and ecological research programs rather than added after an incident (Figure 1).

**Figure 1:**
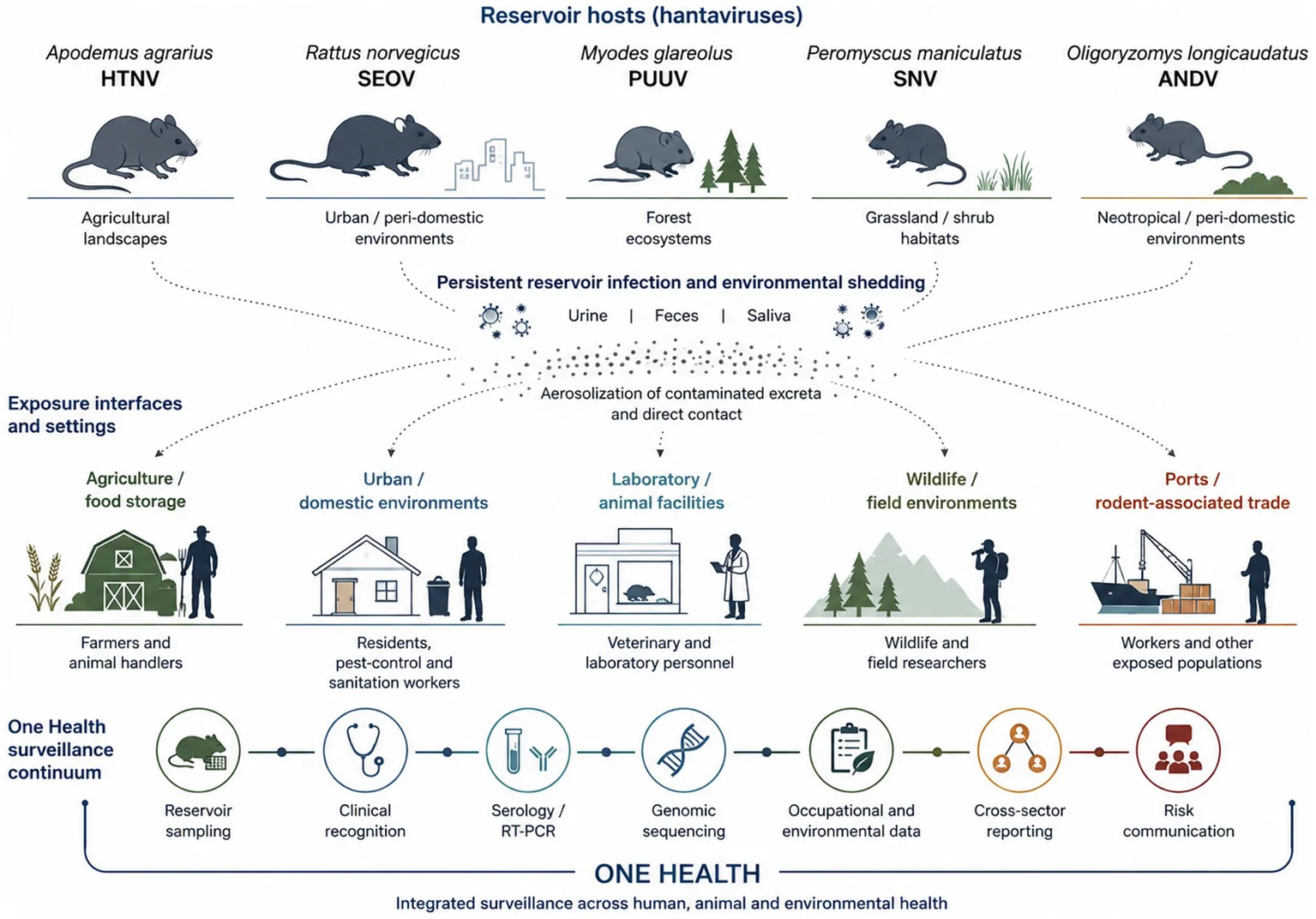
One Health pathways linking representative hantavirus reservoir hosts, exposure interfaces and exposed populations. Representative reservoir-virus associations shown are *Apodemus agrarius* with Hantaan virus (HTNV), *Rattus norvegicus* with Seoul virus (SEOV), *Myodes glareolus* with Puumala virus (PUUV), *Peromyscus maniculatus* with Sin Nombre virus (SNV), and *Oligoryzomys longicaudatus* with Andes virus (ANDV). Infected reservoir rodents may shed virus in urine, feces, and saliva, and human exposure occurs predominantly through aerosolized contaminated material or direct contact across agricultural, domestic, animal-facility, wildlife, and rodent-trade settings. The lower panel summarizes the integrated One Health surveillance functions that link reservoir, clinical, occupational, environmental and genomic information. Original schematic created by the authors.

## 5 Global epidemiology and changing risk landscapes

Hantavirus disease is not uniformly distributed, and incidence trends are difficult to interpret without parallel information on reservoirs and surveillance intensity. The regional summaries below are selective rather than exhaustive, and each is used to illustrate a distinct interpretive problem. Table 3 compares surveillance priorities across epidemiological settings; Table 4 details the data and limitations for each of the human, reservoir, laboratory, and environmental components.

**Table 3:** Risk-proportionate One Health surveillance according to epidemiological context.

Epidemiological setting	Primary surveillance objective	Priority animal and environmental component	Priority human-health component	Program emphasis
Established endemic transmission	Track trends, detect geographic and lineage shifts, evaluate prevention	Longitudinal trapping at fixed sites, species identification, infection prevalence, sequencing, ecological covariates	Standardized notification, laboratory confirmation, severity monitoring	Sustained integrated surveillance and fine-scale risk mapping
Sporadic cases or emerging signal	Determine whether the signal reflects local transmission or a defined exposure network	Targeted trapping around exposure sites and known reservoir habitat	Enhanced testing of compatible febrile, renal or respiratory illness; targeted occupational testing	Rapid investigation, molecular confirmation and sequencing
Animal-facility, pet-rat or feeder-rodent networks	Detect silent reservoir infection before human cases occur	Colony serology, quarantine testing of incoming animals, supply-chain tracing	Symptom-triggered testing of handlers and clinically indicated staff	Veterinary certification, biosafety and traceability
Evidence-sparse or under-surveilled settings	Generate baseline evidence without assuming presence or absence of disease	Sentinel small-mammal surveys at high-contact settings with rigorous species identification	Clinician awareness plus referral testing for selected compatible cases	Small auditable pilots embedded in existing zoonotic or wildlife surveillance
High-connectivity travel and mass-mobility settings	Maintain readiness for imported or travel-associated events	Risk-based rodent and environmental inspection at relevant interfaces	Travel history taking, rapid referral pathways, access to specialized diagnostics	Preparedness, risk communication and international notification links

**Table 4:** Core components of integrated hantavirus surveillance, with the principal limitation of each.

Component	Typical methods	Key metadata	Primary use	Main limitation
Human clinical surveillance	Exposure and travel history, IgM and IgG serology, RT-PCR	Date, place, syndrome, exposure setting, occupation, travel	Case detection, severity monitoring, outbreak investigation	Non-specific presentation and variable access to testing; documented under-notification even in endemic settings
Reservoir surveillance	Live trapping, species identification, serology, RT-PCR on kidney and lung tissue	Species, sex, age class, habitat, coordinates, trapping effort, specimen type	Identify reservoirs, estimate infection prevalence, locate ecological hotspots	Prevalence estimates depend strongly on specimen choice, assay and species identification
Molecular and genomic surveillance	RT-PCR, targeted or whole-genome sequencing, phylogenetics	Sequence quality, host species, date, location, epidemiological links	Lineage tracking, detection of reassortment, source attribution	Requires technical capacity and representative sampling; sequences without metadata have limited value
Occupational surveillance	Task-based exposure assessment, symptom-triggered testing, targeted serosurveys	Job tasks, personal protective equipment used, exposure frequency, setting	Identify high-risk tasks and gaps in prevention	Selection bias and difficulty attributing seropositivity to a single exposure
Environmental information	Rodent indices, land cover and land use, climate, food availability, sanitation indicators	Spatial and temporal resolution, data source and vintage	Risk mapping and targeting of control activity	Ecological associations do not translate directly into individual risk
Cross-sector data integration	Shared case and specimen definitions, interoperable reporting, joint dashboards	Standard time and location fields, agreed data dictionary	Joint situational awareness and coordinated response	Governance, data standards and institutional silos rather than technology

### 5.1 East Asia

HFRS is concentrated in parts of Eurasia, and long-running Chinese surveillance illustrates why declining incidence should not be read as declining risk. Between 2014 and 2023, reported HFRS incidence in China fell from 1.01 per 100,000 to below 0.4 per 100,000, yet hantaviruses were documented across 22 provincial-level administrative divisions, 12 HTNV and 9 SEOV genotypes were identified in circulation, and the area of documented transmission expanded (29). Local studies show the same divergence between human case counts and reservoir circulation. In Xiangyun County, Yunnan, orthohantavirus was detected in 2.72% of 331 rodents sampled alongside clinical cases, with several genotypes circulating in host animals and SEOV predominating among patients (30). In the Republic of Korea, two decades of small-mammal trapping showed high HTNV seropositivity in *Apodemus agrarius* at sites near the Demilitarized Zone and no seropositive animals in one southern province, which supports fine-scale rather than national risk mapping (31). Sequencing has been used prospectively in this setting to link patient exposure sites to infected rodent populations (32).

### 5.2 Europe

European epidemiology is dominated by PUUV and DOBV, with SEOV relevant to ports and urban settings, and is strongly heterogeneous between biomes (7). Climate effects appear to be indirect and regionally variable, mediated through mast years, predator–prey dynamics, winter conditions and land use rather than acting directly on the virus (6, 7). Outbreak investigations expose the practical limits of passive surveillance. During a large PUUV outbreak in Croatia in 2021, 334 cases were notified nationally; among 254 patients managed at a single center, 36.6% reported occupational exposure, cases occurred in areas not previously regarded as endemic, and 14.6% were never notified to public health authorities (33). Even in a setting with established diagnostic services, this degree of under-notification shows why low case counts elsewhere should be interpreted cautiously.

### 5.3 The Americas

HCPS is relatively uncommon in the Americas compared with many severe respiratory infections, but when it occurs it can be fatal, sometimes after a brief period of intense environmental exposure (1, 2). Reservoir studies continue to revise assumptions about host specificity, with SNV detected across multiple sympatric small-mammal species and infectious virus recovered from more than one of them (18). Twenty years of surveillance in Panama illustrate what sustained integrated programs can deliver: disease was shown to be endemic in approximately 27% of the country, corresponding to agroecological conditions that favor the reservoir, with interannual peaks every 4 to 5 years, and decentralization of laboratory testing together with evidence-based case definitions improved notification from primary care and standardized clinical management (34). ANDV remains distinctive within the genus because of documented person-to-person transmission (2).

### 5.4 Southeast Asia, South Asia and other under-surveilled regions

In many under-surveilled regions, limited testing makes it difficult to distinguish low transmission from missed infection. The Southeast Asian meta-analysis noted above pooled 28 studies and estimated hantavirus prevalence of 6.07% in 11,806 small mammals, with higher estimates from enzyme immunoassay than from molecular methods and higher prevalence in rodents than in other small mammals (8). The Cambodian study combining rodent and human sampling found orthohantavirus in 3.3% of rodents and IgG seropositivity in 10.0% of people, with urban residency and flood-prone housing associated with seropositivity (9). Data from Africa remain sparse, and the Madagascar work described above is among a small number of studies applying a structured land-use design (25). In such settings, the highest-value first step is usually targeted reservoir and occupational surveillance with a functioning referral pathway for human testing, rather than a national program.

Three interpretive cautions recur across regions. Apparent emergence often reflects improved diagnostics or geographic expansion of sampling rather than a true change in transmission. Declining human incidence can coexist with stable or increasing viral diversity and persistent reservoir infection. And rodent seroprevalence, which is what most studies report, is a weaker predictor of human disease than the combination of reservoir infection, contamination of used space, and the tasks people perform in it.

## 6 Veterinary public health and occupational exposure

Prevention depends on identifying exposure settings before infection reaches the clinic. The occupational groups of greatest practical interest are farmers, grain and feed workers, animal handlers, veterinarians and veterinary nurses, laboratory-animal and feeder-rodent staff, wildlife biologists, pest-control personnel, sanitation workers, and those who clean rodent-infested buildings (20, 24). Risk is determined less by job title than by task, ventilation, rodent density, cleaning method, and use of respiratory and hand protection. Croatian outbreak data, in which more than a third of hospitalized patients reported occupational exposure, indicate that the occupational contribution is substantial, even when the infection is typically described as environmental (33).

Animal facilities warrant specific veterinary attention because infection there is silent by default. SEOV-infected rats show no clinical signs and shed intermittently, so colony status can only be established by testing. Serological screening detects IgG within approximately 4 weeks of exposure, whereas PCR on blood or tissue may miss animals that are shedding intermittently (23). Recurrent occupational infections at a feeder-rodent breeding farm in Taiwan, where 37.5% of tested rats were seropositive, show the consequence of leaving such testing to chance (35). The practical measures are quarantine and testing of newly introduced animals, periodic colony serology, wet cleaning of cages rather than dry sweeping, documented exposure records, and a named referral pathway for symptomatic staff (20, 23, 26).

At the community level, rodent control, sanitation, sealed feed storage, structural exclusion, and safe cleaning reduce exposure and simultaneously create surveillance opportunities, provided that a standardized subset of captured animals is identified, sampled, and georeferenced (28). In most low-capacity settings, this is the least expensive route to reservoir data because the trapping effort already exists; only sampling, identification, and data capture need to be added.

Occupational messaging is more effective when it targets tasks rather than the general presence of rodents. Advice to wet down material before cleaning, to avoid dry sweeping and high-pressure washing in enclosed infested spaces, to use appropriate respiratory protection during specified tasks, and to report exposures promptly, is actionable in a way that generic warnings about rodents are not.

## 7 Diagnostics, laboratory networks and genomic surveillance

Clinical diagnosis begins with exposure history and a compatible syndrome, but laboratory confirmation is essential because hantavirus disease overlaps with many febrile, renal, hemorrhagic and respiratory illnesses (2, 3, 36). Serology remains the most widely used approach, especially IgM and IgG testing. Molecular detection is most useful during the brief viremic phase, while sequencing can help identify lineage during epidemiological investigations (32). Assay performance varies by platform and virus species, and cross-reactivity means a positive pan-hantavirus test may identify the group without resolving the exact species.

Specimen choice is equally important when rodents are tested. In naturally infected rodents from Hungary, viral RNA was detected in all kidney samples from PCR-positive animals but in only 55% of lung samples; only 3 of 20 urine samples were positive, and five animals were seropositive with no detectable RNA in any tested organ (37). Two practical conclusions follow. Sampling a tissue with lower diagnostic sensitivity can underestimate infection prevalence, and serological and molecular results measure different things and should be reported separately rather than combined into a single positivity figure.

Sampling should be paired with accurate host identification, capture location, habitat description, trapping effort, and date. Without these, positive results have limited value for spatial risk assessment or for comparison across studies (13). Species misidentification is a recurring and under-acknowledged source of error, and it matters more now that virus-host associations are known to be less exclusive than was once assumed (18).

Environmental sampling of dust, bedding, or surfaces can supplement surveillance in defined high-risk settings, but methods are considerably less standardized than those for direct animal testing, and detection is influenced by substrate, temperature, and time since contamination. Negative environmental results should not be interpreted as evidence that local rodents are uninfected, and a single positive rodent does not by itself establish sustained human disease risk.

Laboratory capacity is best designed as a network rather than replicated everywhere. Local and regional laboratories can perform validated screening assays; reference laboratories provide confirmatory testing, sequencing, assay validation, and complex serology. Biosafety requirements depend on the procedure and on whether an infectious virus is propagated; most diagnostic workflows do not require virus culture. The Panamanian experience, in which decentralizing a defined test while retaining central reference functions improved notification and case management, is a useful model (34). The value of referral networks is equally evident in reverse: during the 2026 ANDV event, confirmation of a case on Tristan da Cunha was delayed until a specimen could be shipped to the United Kingdom because no local capacity existed (10).

## 8 A risk-proportionate, tiered One Health surveillance framework

Recommending identical surveillance everywhere is neither affordable nor epidemiologically justified. We therefore propose a framework built on five shared pillars that are scaled by tier rather than replaced between tiers: reservoir surveillance; clinical recognition and laboratory access; molecular and genomic characterization; occupational and environmental risk assessment; and cross-sector data integration (11, 12, 38). What distinguishes this framework from previous descriptive One Health syntheses is that each tier carries an explicit entry trigger, a stated surveillance objective, a defined minimum dataset, and criteria for both escalation and de-escalation. The framework is an evidence-informed operational proposal rather than a validated surveillance standard, and its triggers have not been tested against outcome data. Descriptive reviews generally recommend integration without specifying the point at which a setting should do more, or less.

Tables 5, 6 summarize the tier transitions and show how each of the five shared pillars changes with surveillance intensity. Across all tiers, confirmatory testing remains accessible through a named referral route. Sequencing at Tier 2 is directed toward informative detections with adequate specimens and epidemiological links; Tier 3 sets a locally feasible routine sampling fraction. No universal prevalence percentage or fixed sequence count is proposed as an escalation threshold. A single unconfirmed positive finding prompts verification and exposure assessment before a tier decision.

**Table 5:** Tiered escalation and de-escalation framework for One Health hantavirus preparedness.

Tier	Entry trigger	Objective	Core components	Escalation indicators	De-escalation or adaptation
Tier 1: baseline readiness	No recognized local transmission, low reported incidence, or no reservoir data	Establish whether a signal exists and create the means to detect one	Sentinel small-mammal sampling at high-contact settings; species identification and minimum metadata; clinician awareness; named referral pathway; occupational education	Repeated animal positives, a confirmed compatible human infection, or a consistent high-risk exposure cluster	Not applicable; Tier 1 is the maintained baseline
Tier 2: enhanced surveillance	Sporadic cases, local animal positivity, occupational cluster, or unexplained epidemiological signal	Determine whether the signal reflects established transmission, a restricted focus, or a specific exposure network	Expanded case finding; targeted occupational testing; repeated seasonal trapping; confirmatory molecular diagnostics; sequencing; exposure and land-use mapping	Transmission sustained across seasons, geographic spread, rising confirmed case counts, or repeated related lineages	Return to Tier 1 with referral retained after a locally defined observation period without further detections, provided the initiating signal has been adequately investigated
Tier 3: sustained integrated surveillance	Established endemic transmission or a persistent high-risk focus	Move from case counting to prediction, targeting and evaluation	Longitudinal reservoir monitoring at fixed sites; routine genomic surveillance with metadata; standardized notification with severity; ecological covariates; evaluated prevention programs	Not applicable; Tier 3 is the highest level	Redirect effort when case geography, lineage distribution, severity, reservoir abundance or seasonal pattern changes materially

**Table 6:** Scaling of the five shared surveillance pillars across the proposed tiers.

Pillar	Tier 1: baseline readiness	Tier 2: enhanced investigation	Tier 3: sustained surveillance
Reservoir	Named sentinel sites; species, trapping effort, season and assay recorded.	Repeated sampling at implicated and comparison sites across relevant seasons.	Fixed-site trends with documented denominators and regular review of species and geography.
Clinical and laboratory	Clinician awareness and a named confirmatory referral route.	Active case finding using an agreed case definition and prompt confirmatory referral.	Routine reporting, severity trends, diagnostic quality assurance and referral for unusual cases.
Molecular and genomic	Reference laboratory access for confirmed unusual findings; no routine sequencing requirement.	Sequence informative human or animal detections when specimens and epidemiological links permit.	Sequence a predefined, locally feasible subset of confirmed detections with standard metadata.
Occupational and environmental	Identify high-contact tasks and provide safe cleaning and handling advice.	Investigate specific exposures, sites, protective practices and land-use context.	Repeat task-based risk assessments and evaluate prevention measures over time.
Cross-sector integration	Named human, animal and laboratory contacts; shared minimum fields.	Joint case and reservoir investigation with a documented decision to retain or change tier.	Regular linked-data review, feedback to participating sectors and periodic program evaluation.

For planning, a confirmed local human case or a confirmed animal detection with a plausible exposure route could initiate Tier 2; a persistent cluster across seasons or verified spread beyond the initial focus could justify Tier 3. These signals are suggestive decision points, not sensitivity- or specificity-tested cutoffs. A return to Tier 1 requires documented investigation and observation over the locally relevant reservoir season. A new signal reopens the decision immediately.

### 8.1 Tier 1: baseline readiness in evidence-sparse or low-incidence settings

Tier 1 applies where there is no recognized local transmission, low reported incidence, or no reservoir data at all. The objective is to establish whether a signal exists and to create the means of detecting one, not to estimate national prevalence. The components are a small sentinel network at high-contact settings such as grain and feed stores, live animal markets, laboratory and breeding colonies, and urban waste sites; standardized trapping with reliable species identification and a minimum metadata set; serology or PCR on a defined subset of animals; clinician awareness of the compatible syndromes; and a named referral pathway for confirmatory human testing. Wherever possible, these functions should be attached to existing zoonotic disease, wildlife health, laboratory-animal, or municipal rodent-control systems rather than created separately (14, 28). The deliverable is a reproducible signal-detection platform with an auditable denominator. Escalation is warranted by repeated animal positives at one or more sites, a confirmed compatible human infection, or a consistent cluster of high-risk exposures.

### 8.2 Tier 2: enhanced surveillance after sporadic or emerging signals

Tier 2 is triggered by a confirmed sporadic human case, a confirmed animal detection with a plausible exposure pathway, an occupational cluster, or another credible epidemiological signal. At this stage, the main question is whether the signal reflects ongoing local transmission, a limited geographic focus, or a particular exposure network. Components include expanded case finding under a standardized case definition; targeted serosurveys and symptom-triggered testing in the implicated occupational group; repeated trapping across seasons at and around the exposure site; confirmatory molecular diagnostics; and sequencing sufficient to establish whether human and animal detections are related. Land-use and environmental data become materially useful at this stage because they can direct control activity rather than only describe risk (6). Escalation is indicated by transmission sustained across seasons, spread beyond the initial focus, rising confirmed case counts, or repeated detection of related lineages.

De-escalation should be equally explicit, although no validated threshold exists and none is proposed here. De-escalation may be considered after a locally defined observation period without additional human or animal detections, provided that the initiating signal has been adequately investigated and that ongoing surveillance gives no indication of persistent transmission. The length of that period should be set locally based on reservoir seasonality, trapping effort, and diagnostic capacity, rather than adopted from another setting. Treating de-escalation as a deliberate decision is important because, otherwise, enhanced surveillance can persist out of habit or, at the other extreme, be stopped before the available evidence supports it.

As an illustrative operational schedule, the responsible agencies could review Tier 2 signals every 3 months and again after the next locally relevant reservoir season, documenting testing intensity and whether the initiating exposure continues. A return to Tier 1 would require an adequately investigated source and no further confirmed signal during that observation window. Following de-escalation, the sentinel team should reassess at least annually and promptly after a new human case, an animal detection, an occupational cluster, or a major ecological change. These intervals are suggestions for planning, not validated thresholds; a local authority should set the final schedule.

### 8.3 Tier 3: sustained surveillance where transmission is established

Tier 3 applies where transmission is established, or a persistent high-risk focus is known. The emphasis shifts from counting cases to anticipating changes in risk, directing control, and evaluating prevention. Surveillance includes repeated reservoir sampling at fixed sites, genomic analysis linked to host and spatial metadata, standardized clinical reporting of severity, ecological variables, fine-scale spatial analysis, and evaluation of prevention measures. Long-term programs in China and the Republic of Korea have shown expanding affected areas despite falling incidence and stable local hotspots at sub-provincial scale (29, 31). At this level, changing conditions usually call for redirecting surveillance rather than moving to another tier. Indicators warranting reassessment include a shift in the geographic distribution of cases, a change in lineage distribution or the appearance of reassortants, a change in severity, a marked change in reservoir abundance, or an outbreak pattern inconsistent with the established seasonal profile.

### 8.4 Requirements common to all tiers

For each tier, the five pillars require a common minimum set of records: date and location, host or patient identification, exposure setting, collection and assay details, result, reporting route, and response decision. At Tier 1, a reference laboratory remains reachable for confirmation of unusual findings. At Tier 2, sequencing is considered when paired specimens and assay quality permit a meaningful comparison. At Tier 3, a predefined sampling fraction and quality criteria support routine trend monitoring. Sequencing volume cannot be prescribed universally because case counts, specimen quality, and laboratory capacity differ. Neither a rodent prevalence percentage nor an isolated seropositive animal should automatically trigger a tier change without confirmation and exposure assessment.

Four requirements are constant across tiers. The first is standardization: shared case definitions, specimen metadata standards, and laboratory quality assurance, without which data cannot be pooled across sectors or borders (13). The second is referral. Every tier requires a functioning route to confirmatory testing, and this component most often fails; its failure converts a rare imported case into a delayed diagnosis (10, 39). The third is data interoperability: human case data, animal results, sequences, and environmental indicators should use compatible time and location fields, and even a simple shared dashboard improves situational awareness when it is supported by agreed definitions and governance (38, 40, 41). Advanced analytical methods, including machine learning, are unlikely to add much until these basic data structures are in place (41). The fourth requirement is proportionate risk communication. Messages should neither amplify an unconfirmed local threat nor dismiss rodent-borne risk because confirmed cases are uncommon. Communication is most useful when it explains where exposure occurs, which clinical features should prompt assessment, and how to safely clean contaminated areas.

Tier 1 is intended to be feasible in resource-limited settings. It does not require a dedicated laboratory, continuous trapping, or a new institution. A workable baseline includes defined sentinel sites, a common minimum dataset, modest annual sampling, trained staff, and a written referral arrangement with a reference laboratory. When added to existing rodent-control or wildlife-health programs, the additional costs are primarily for sample transport, testing, and data management. Costs at higher tiers are then incurred only when evidence warrants them, which is the central practical claim of the framework.

## 9 The 2026 multi-country Andes virus event as a preparedness test

The World Health Organization was notified on 2 May 2026 of severe acute respiratory illness among passengers and crew of the expedition ship M/V Hondius, which had departed from Ushuaia, Argentina, on 1 April. As of 2 July, WHO reported 13 cases, including 12 laboratory-confirmed and one probable case, and three deaths. Contact follow-up occurred in 33 countries and overseas territories, with 317 high-risk contacts completing quarantine and monitoring and 336 low-risk contacts completing self-monitoring. WHO reported that the initial infection was likely acquired on land before embarkation and that subsequent person-to-person transmission occurred aboard the vessel, while the original source remained undetermined (10).

Several features are instructive for preparedness rather than for epidemiological interpretation. The diagnosis was reached only after the differential was widened to include HCPS in patients with a suggestive travel history, and confirmation depended on a reference laboratory in a third country (10, 39). A case on Tristan da Cunha was initially classified as probable; limited local diagnostic capacity delayed confirmation until a sample was tested in the United Kingdom, while early detection and isolation prevented onward transmission (10). Contact management across more than 30 jurisdictions required guidance that had to be produced under time pressure (42, 43). Finally, the ANDV-specific possibility of person-to-person transmission made infection control relevant in a way that would not apply to most hantaviruses (2).

The event should not be read as evidence that hantavirus risk has become globally widespread. Transmission remained confined to a defined exposure network, no secondary transmission was reported in receiving countries, and the endemic distribution of ANDV is unchanged. Its value is as a stress test of the components emphasized throughout this review: exposure history taking, access to referral diagnostics, and coordinated communication. For the great majority of hantaviruses, prevention remains anchored at the reservoir-human interface rather than in contact management.

### 9.1 Infection prevention and contact management for suspected Andes virus

For suspected or confirmed ANDV infection, healthcare facilities should promptly isolate the patient, notify infection prevention and public health teams, and assess staff and other close contacts. In its May 2026 advisory, CDC recommended an airborne infection isolation room and a gown, gloves, eye protection, and an N95 or higher-level respirator for personnel entering the room (42). WHO recommended risk-based identification and monitoring of contacts following the cruise-ship event (44). The applicable isolation duration and follow-up procedures must follow current local guidance and individual exposure assessment; the 42-day maximum incubation interval is relevant to contact follow-up, not a universal patient isolation duration (42, 44). A referral route for diagnostic specimens and named responsibility for contact tracing should be agreed before an event (10, 42, 44).

## 10 Discussion

### 10.1 What the integrated evidence indicates

The material reviewed here supports a specific reading. Hantavirus risk is generated almost entirely outside health services, is highly focal, and is shaped by determinants that are measurable in advance: reservoir abundance and species composition, land use, housing and storage conditions, and work practice. It also indicates that the quantities most often measured are not the quantities that most directly predict human disease. Rodent seroprevalence is the commonest reported metric, but the Hungarian tissue comparison shows that measured prevalence depends heavily on specimen choice (37), and the New Mexico data show that it depends on which species are sampled (18). Meanwhile the determinants that translate reservoir infection into human infection, namely contamination of used space and the tasks people perform within it, are rarely measured at all. This mismatch is the main reason why reservoir data have not been more useful operationally.

### 10.2 What the proposed framework adds

A retrospective application to the 2021 Croatia outbreak illustrates the decision logic without validating it. A setting with established endemic transmission would start in Tier 3, while cases detected outside previously recognized endemic districts would prompt targeted investigation in those areas, including exposure histories, confirmatory testing, and reservoir sampling. The observed under-notification would also warrant an audit of clinical notification (33). Panama offers a second qualitative check: two decades of reported endemic transmission and repeated peaks support sustained Tier 3 monitoring and laboratory decentralization (34). Neither published report supplies the site-level time series, trapping denominators, and prospective decision records needed to calculate sensitivity, specificity, or the costs of proposed triggers.

Existing One Health syntheses reliably conclude that integration is needed (11, 12, 38). Fewer specify the decision rules. The framework proposed here contributes three things beyond restating the case for integration. It attaches explicit entry triggers and escalation criteria to each level, so that a setting can determine which tier it currently occupies and what would move it. It defines a minimum credible program for settings with no current evidence, which is the situation of most under-surveilled regions and the one least well served by existing guidance. And it treats de-escalation as an explicit option, which matters because surveillance that cannot be scaled down is rarely scaled up. None of these components is individually novel; the contribution is their assembly into decision rules that can be applied without first knowing the local burden.

### 10.3 Implementation

Implementation is most realistic where the framework is attached to functions that already exist. Municipal rodent control, laboratory-animal health monitoring, wildlife health surveillance and market or abattoir inspection all generate contact with the reservoir population and can supply samples and denominators at marginal cost (14, 28). The harder requirements are institutional rather than technical: a case definition shared between human and animal health services, a written referral agreement with a reference laboratory, and a named individual in each sector responsible for acting on a signal. The 2026 event highlighted these organizational gaps more clearly than any lack of laboratory technology (39, 43).

### 10.4 Matching surveillance effort to available resources

Feasibility can be illustrated with a planning scenario rather than a transferable cost claim. If one field visit requires two staff for 1 day and yields 20 specimens, a Tier 1 pilot with two sites visited twice annually involves four visits, eight field person-days, and about 80 specimens. A Tier 2 investigation at four sites visited four times involves 16 visits, 32 field person-days, and about 320 specimens. A Tier 3 network of eight sites visited four times involves 32 visits, 64 field person-days and about 640 specimens per year. These are arithmetic examples, not recommended sample sizes or estimates from the cited studies. They exclude travel time, confirmatory testing, sequencing, training and data management; each jurisdiction should price these components and adjust the design to seasonality, expected yield and available staff.

The case for proportionality is not simply about saving money. In low-prevalence settings, uniform surveillance can consume scarce capacity while producing data that are difficult to interpret. For example, a national rodent serosurvey in a country with no confirmed human cases and no reference laboratory may yield a prevalence estimate without providing a clear route to assess human risk or act on the finding. A smaller sentinel network, built around reliable species identification and a functioning referral pathway, can use the same resources to detect a signal and assess its credibility. Proportionality also protects credibility, since programs that generate findings without a defined response tend not to survive the next budget cycle.

### 10.5 Surveillance inequality and the interpretation of missing evidence

The most consequential interpretive error in this field is treating the absence of reports as evidence of absence. Two findings in this review help bound the problem. In Croatia, a setting with established diagnostic capacity and a recognized endemic zone, 14.6% of hospitalized patients in a large outbreak were never notified to public health authorities, and cases occurred in districts not previously considered endemic (33). In Southeast Asia, pooled small-mammal prevalence of 6.07% coexists with limited human diagnostic recognition (8), and in Cambodia human seropositivity of 10.0% was documented in a country with essentially no reported clinical burden (9). If under-ascertainment of that magnitude occurs where capacity exists, inference from silence where capacity does not exist is unsafe. The defensible statement about an evidence-sparse setting is that its status is unknown, and the appropriate response is a Tier 1 program designed to resolve it.

### 10.6 Pressures on the human-animal-environment interface

Under plausible future climate scenarios, changes in precipitation, temperature and vegetation may alter mast production, rodent survival and the timing of contact with people. A mast year may be followed by an increase in rodents, yet its effect on human disease depends on species, habitat, housing, and behavior. Surveillance programs should compare seasonal trapping and human diagnoses with local ecological observations and review sentinel locations after unusual mast, flooding, drought or land-use change, without assuming the same directional effect across regions (5–7).

Several pressures are likely to alter exposure patterns, although the direction and magnitude are setting-specific and should not be asserted as general rules. Land-use change has the clearest empirical support, with agricultural expansion associated with increased risk in parts of Asia and South America and forest cover associated with incidence in Europe, while associations with urbanization differ in direction across studies (6). Climate influences appear to operate indirectly, through mast years, winter severity, predator–prey dynamics and reservoir density, rather than through any direct effect on the virus (5, 7). Urbanization changes rather than removes risk, because commensal Rattus populations persist in cities, ports, food-storage systems and trade networks (20, 26). International mobility and trade in laboratory and companion rodents create exposure networks that cross jurisdictions faster than surveillance data do (10, 20). Food-storage practice and waste management remain among the most modifiable determinants and among the least studied.

### 10.7 Where the evidence is inconsistent or incomplete

Several areas warrant explicit caution. Reported rodent prevalence estimates are not comparable across studies, because trapping effort, season, specimen type, assay and species identification all differ, and the resulting heterogeneity is large enough to dominate genuine geographic variation (13, 37). The relationship between climate variables and human incidence is inconsistent between regions and between studies within regions, and much of the supporting work is ecological in design and cannot support causal inference at the individual level (6, 7). Several non-primary small-mammal species can harbor and shed infectious virus, but their contribution to human exposure remains unknown (18). The performance of serological assays across virus species is variable and is rarely evaluated in the settings where it matters most. And the effectiveness of specific preventive measures, including respiratory protection during defined tasks, wet cleaning and structural exclusion, rests largely on plausibility rather than on intervention studies.

### 10.8 Research priorities

Five priorities follow directly from these gaps. First, harmonized minimum reporting standards for reservoir studies, covering trapping effort, specimen type, species identification method, assay and metadata, would do more for global synthesis than additional prevalence surveys (13). Second, prospective studies linking reservoir infection, environmental contamination, measured human behavior and confirmed human outcomes are needed to establish which reservoir metrics actually predict risk. Third, occupational cohorts among animal handlers, feeder-rodent and laboratory staff, field biologists, sanitation and pest-control workers and agricultural workers would identify the specific tasks carrying the greatest risk and would permit evaluation of protective measures (24, 35). Fourth, genomic surveillance should be routinely coupled to host and spatial metadata, with quality-controlled and open sequence sharing, since sequences without metadata cannot support source attribution (16, 32). Fifth, implementation research is needed on governance, interoperability, sustainability and cost, because the technical components of integrated surveillance are far better described than the conditions under which they are sustained (12, 38, 40, 41).

### 10.9 Strengths and limitations

This review integrates clinical, reservoir, occupational, laboratory and surveillance evidence into a locally adaptable framework. Its narrative design limits inference: no systematic protocol, prospective screening record or formal risk-of-bias assessment was used, and study selection may be biased. Evidence is uneven across regions, and differences in assays, case definitions, trapping protocols, and host identification complicate comparisons. Rodent infection does not quantify human risk, and prospective occupational and implementation cost data are scarce. The Croatia and Panama examples illustrate tier decisions but do not validate the triggers. Suggested workloads, reassessment intervals, and sequencing choices need local testing. Prospective evaluations should prespecify signals and sampling, then measure case ascertainment, response time, missed signals and resource use before any thresholds are adopted as policy.

## 11 Conclusion

Hantavirus preparedness begins before a patient reaches clinical care. In evidence-sparse settings, a small sentinel network with reliable host identification, task-based prevention and access to confirmatory testing offers a concrete starting point. A credible case or animal finding should trigger a defined investigation; established transmission warrants sustained, linked human and reservoir surveillance. These actions depend on shared records and clear responsibility across sectors. The three-tier framework is intended to help local teams plan and review those decisions. Prospective testing must establish whether its suggested triggers improve detection, response time and efficient use of resources before they are adopted as fixed policy.
